# Association between female genital mutilation type and lower urinary tract symptoms among symptomatic Somali women: a cross-sectional study

**DOI:** 10.1136/bmjopen-2026-118031

**Published:** 2026-09-02

**Authors:** Umut Erkok, Esra Keles, Adil Barut, Bilgehan Saglik, Levent Aksoy, Mohamed Omar Hirsi, Said Mohamed Mohamud, Zeynep Omer, Gizem Kul, Meryem Ceylan

**Affiliations:** 1 Obstetrics and Gynecology Department, Somalia Mogadishu Turkey Recep Tayyip Erdogan Training and Research Hospital, Mogadishu, Banaadir, Somalia; 2 Obstetrics and Gynecology Department, Kartal Dr Lutfi Kirdar City Hospital, Istanbul, Türkiye; 3 Division of Maternal-Foetal Medicine, Department of Obstetrics and Gynaecology, Cerrahpasa Faculty of Medicine, Istanbul University-Cerrahpasa, Istanbul, Türkiye; 4 Division of Gynaecology Oncology, Department of Obstetrics and Gynaecology, Cerrahpasa Faculty of Medicine, Istanbul University-Cerrahpasa, Istanbul, Türkiye; 5 Somalia Mogadishu Turkey Recep Tayyip Erdogan Training and Research Hospital, Mogadishu, Somalia; 6 Obstetrics and Gynaecology Department, Istanbul Bakirkoy Dr Sadi Konuk Training and Research Hospital, Istanbul, Turkey; 7 Obstetrics and Gynaecology Department, Diyarbakir Gazi Yasargil Training and Research Hospital, Diyarbakır, Türkiye; 8 Obstetrics and Gynaecology Department, Lokman Hekim University, Ankara, Türkiye

**Keywords:** Postpartum Women, SEXUAL MEDICINE, Sexual dysfunction

## Abstract

**Abstract:**

**Background:**

Female genital mutilation (FGM)/cutting remains highly prevalent in Somalia and is associated with a range of long-term gynaecological and urological complications. However, data on lower urinary tract symptoms (LUTS) and their severity across different FGM types are limited. The aim of this study was to examine the association between FGM type and LUTS among symptomatic care-seeking women with FGM and to evaluate whether symptom severity differed according to FGM type.

**Design:**

This cross-sectional study included 122 symptomatic women with a history of FGM who presented with LUTS between January and April 2024.

**Methods:**

Sociodemographic characteristics, FGM type, clinical symptoms, and vaginal and urine culture results were recorded. LUTS severity and associated bother were assessed using the International Consultation on Incontinence Questionnaire–Female Lower Urinary Tract Symptoms Long Form (ICIQ-FLUTS-LF). Comparisons were made between women with FGM types I–II and those with type III.

**Results:**

The mean age of participants was 31.7±7.6 years. Women with FGM type III had a significantly higher prevalence of positive urine culture growth, history of urinary tract infection (UTI) and prior antibiotic use, and abdominal pain compared with those with types I–II (p<0.05). Median scores for all LUTS domains and corresponding bother scores were significantly higher in women with FGM type III compared with types I–II (all p<0.001), and similar findings were confirmed in regression analyses.

**Conclusion:**

Among symptomatic care-seeking women with FGM, type III was associated with greater LUTS burden, higher reported history of UTI and greater symptom-related distress compared with types I and II.

**STRENGTHS AND LIMITATIONS OF THIS STUDY**

- This study used a standardised and validated instrument (International Consultation on Incontinence Questionnaire–Female Lower Urinary Tract Symptoms Long Form) to comprehensively assess lower urinary tract symptoms and associated bother.
- Female genital mutilation (FGM) type classification was based on clinical assessment, improving the accuracy of exposure categorisation.
- The cross-sectional design limits the ability to establish temporal or causal relationships between FGM types (I–II and III) and urinary symptoms.
- The study population consisted of symptomatic care-seeking women with FGM, which may limit the generalisability of the findings to asymptomatic women with FGM or the broader community.
- Self-reported information on urinary tract infection history and previous antibiotic use may have been subject to recall bias.

## Introduction

 Female genital mutilation (FGM) comprises all procedures involving the partial or total removal of external female genitalia or other injury to the female genital organs for non-medical reasons. This practice is estimated to affect approximately 200 million women and girls globally.^1^ Since the initial 1997 WHO categorisation, the practice has been systematically classified into four primary types to facilitate precise clinical reporting and epidemiological study. Type I involves partial or total removal of the clitoral glans and/or prepuce, with or without excision of the clitoris (clitoridectomy), while type II (excision) includes the removal of the glans and labia minora. Type III (infibulation) represents the most invasive form, involving the narrowing of the vaginal orifice through the creation of a covering seal formed by repositioning the labia. Type IV encompasses all other deleterious non-medical procedures, including piercing, scraping and cauterisation.^2^ Despite ongoing multi-level efforts to eradicate it, the practice remains prevalent across numerous regions, most notably within sub-Saharan Africa, with Somalia continuing to report one of the highest prevalence rates globally.^3^ FGM is associated with various complications, such as severe pain, haemorrhage and acute infections, alongside long-term sequelae, including obstetric complications, sexual dysfunction and urological disorders.^4–6^ The anatomical alterations and extensive scarring associated with FGM can obstruct urinary flow, precipitate urinary retention and create an environment conducive to bacterial proliferation.^7–9^ Among urological complications, recurrent urinary tract infections (UTIs) represent a significant clinical burden; previous studies have established a heightened risk of UTIs in women who have undergone FGM.^10 11^

Understanding the clinical impact of FGM on urological health is important for informing the development of appropriate screening protocols and treatment strategies. Somalia’s healthcare system faces distinct challenges in managing FGM-related complications, including limited access to specialist services and persistent cultural barriers to healthcare-seeking behaviour.^12^ Although the 2025 WHO recommendations support screening and clinical evaluation of women living with FGM-related complications.^13^ Furthermore, the limited availability of comprehensive data on lower urinary tract symptoms (LUTS), UTIs and associated risk factors among Somali women who have undergone FGM may restrict evidence-based clinical decision-making and individualised patient management. Therefore, the aim of this study was to examine the association between FGM type and LUTS among symptomatic care-seeking women with FGM and to evaluate whether symptom severity differed according to FGM type.

## Materials and methods

### Study design and participants

This cross-sectional study included 122 symptomatic women who presented to the Department of Gynaecology at Mogadishu Somali Turkey Training and Research Hospital in Mogadishu, the capital city of Somalia, between 1 January 2024 and 1 April 2024. Data collected included age, type of FGM, parity, marital status, area of residence, occupation, educational status, family income, the person who performed the FGM procedure and results of vaginal and urine cultures.

All participants were asked to complete the International Consultation on Incontinence Questionnaire–Female Lower Urinary Tract Symptoms Long Form (ICIQ-FLUTS-LF). For participants who were illiterate, the questionnaire was administered by a gynaecology specialist (MOH or UE) through face-to-face interviews, with responses recorded according to the statements of participants. All procedures were performed by two specialist physicians.

The inclusion criteria were women aged ≥18 years presenting with LUTS. Exclusion criteria included age <18 years, pregnancy, psychiatric disorders requiring pharmacological treatment, use of medications known to affect lower urinary tract function (such as anticholinergics, alpha-blockers or diuretics), comorbid conditions (including renal or liver failure, diabetes mellitus, hypertension and coronary artery disease) and refusal to participate in the study. Consequently, the study population comprised a selected group of symptomatic, care-seeking women with FGM who met the predefined eligibility criteria.

### Questionnaire

The ICIQ-FLUTS-LF is a validated and comprehensive instrument used to assess the severity of LUTS and their impact on health-related quality of life in women. The questionnaire consists of 18 items, each comprising two components: symptom severity, rated on a 5-point Likert scale from 0 (no symptoms) to 4 (maximum severity), and symptom-related bother, assessed on a numeric scale from 0 (not bothersome) to 10 (extremely bothersome).

The ICIQ-FLUTS-LF covers all major LUTS domains, including storage symptoms (urgency, frequency and nocturia), voiding symptoms (hesitancy, weak stream and straining), urinary incontinence (stress and urgency incontinence), bladder pain (discomfort during bladder filling or voiding), post-micturition symptoms (sensation of incomplete emptying and post-void dribbling) and voluntary interruption of urine flow. Both symptom severity and associated bother scores are recorded for each domain, allowing a comprehensive evaluation of the functional impact and quality-of-life burden of LUTS.^14^

### Clinical examination and laboratory investigations

All participants underwent a standardised urogynaecological evaluation, including medical history taking, physical examination, stress incontinence testing, pelvic ultrasound, urinalysis and vaginal and urine cultures. Urine specimens were collected using midstream clean-catch technique following standardised protocols to minimise contamination risk. Microbiologic analysis was performed using standard laboratory techniques. Urine cultures were considered positive when bacterial growth exceeded 10⁵ colony-forming units (CFU)/mL for clean-catch specimens. Bacterial identification and antimicrobial sensitivity testing were conducted according to Clinical and Laboratory Standards Institute guidelines. All clinical examinations and sample collections were performed under sterile conditions in accordance with standard clinical protocols. Patient privacy, confidentiality and dignity were strictly maintained throughout all study procedures.

### Patients and public involvement

Patients and the public were not involved in the planning, conduct or reporting of this study, despite its prospective design, due to logistical and contextual constraints during the study period. There are no plans to disseminate the results directly to study participants or the wider patient community. However, the findings are being shared within relevant professional communities in Somalia and with policymakers, with the aim of informing future health policy decisions.

### Data processing and analysis

Statistical analysis was performed using SPSS V.30.0 (IBM Corp, Armonk, New York, USA). The normality of continuous variables was assessed using the Kolmogorov–Smirnov test and visual inspection of histograms. Continuous variables were presented as mean±SD or median (IQR), as appropriate. Categorical variables were summarised as frequencies and percentages. Comparisons between groups were performed using the Mann–Whitney U test for non-normally distributed continuous variables and the χ^2^ test for categorical variables, as appropriate. Separate univariable logistic regression analyses were initially performed to estimate crude ORs with 95% CIs. Variables showing potential associations (p<0.20) in the univariable analyses were considered candidate covariates for the corresponding multivariable logistic regression models. This threshold was used as a pragmatic screening criterion to construct parsimonious models while reducing the risk of model instability given the available sample size. Adjusted ORs and 95% CIs were calculated from separate multivariable logistic regression models constructed for each LUTS domain. All statistical tests were two-tailed, and a p value <0.05 was considered statistically significant.

All eligible women who met the predefined inclusion criteria during the study period were included in the final analysis. The regression analyses included 113 participants, comprising 64 women with FGM types I–II and 49 women with FGM type III. Women with FGM type IV were excluded from these comparative analyses.

## Results

### Participant characteristics

A total of 122 symptomatic women with FGM were included in the final analysis. The mean age of the participants was 31.7±7.6 years (range 19–58 years). The median age at FGM was 6 years (range 1–9 years). FGM type II was the most prevalent, affecting 41.0% (n=50) of participants, followed by type III in 40.2% (n=49). FGM type I was identified in 11.5% (n=14) of participants, while type IV accounted for 7.4% (n=9). The majority of FGM procedures were performed by traditional circumcisers (63.1%, n=77), whereas trained midwives or nurses performed 35.2% (n=43) of procedures (table 1).

**Table 1:** Demographic, obstetric and sociodemographic characteristics of participants

Parameters	n=122
Age (years), mean±SD	31.7±7.6
Number of live births, median (range)	4 (0–15)
Number of abortions, median (range)	0 (0–7)
Number of stillbirths, median (range)	0 (0–11)
Age at FGM (years), mean±SD	5.5±1.6
Marital status, n (%)	
Married	116 (95.1)
Divorced	6 (4.9)
Education level, n (%)	
No formal schooling	45 (36.9)
Primary education	23 (18.9)
Secondary education	37 (30.3)
University level education or higher	17 (13.9)
Residence, n (%)	
Urban	106 (86.9)
Rural	16 (13.1)
Occupation, n (%)	
Housewife	107 (87.7)
Private employee	8 (6.6)
Daily labourer	4 (3.3)
Government employee	3 (2.5)
Annual household income, n (%)	
Income equals expense	88 (72.1)
Income exceeds expense	18 (14.8)
Income less than expense	16 (13.1)
Type of FGM, n (%)	
Type I	14 (11.5)
Type II	50 (41.0)
Type III	49 (40.2)
Type IV	9 (7.4)
FGM practitioner, n (%)	
Traditional circumciser	77 (63.1)
Trained midwife/nurse	43 (35.2)
Traditional birth attendant	1 (0.8)
Other traditional	1 (0.8)

FGM, female genital mutilation.

The distribution of microorganisms in vaginal and urine cultures is presented in table 2. In both sample types, the most common finding was no growth or non-pathogenic flora. In vaginal cultures, the most frequently isolated microorganisms were *Escherichia coli* (5.8%) and *Candida* spp (4.1%). In urine cultures, *E. coli* (9.0%) and *Klebsiella* (2.5%) were the most commonly identified pathogens.

**Table 2:** Vaginal and urine culture results

Culture type	Microorganism	n=122 (%)
Vaginal	No growth or non-pathogen	100 (82.6)
	*Escherichia coli*	7 (5.8)
	*Candida* spp	5 (4.1)
	*Klebsiella*	5 (4.1)
	Diphtheroid	2 (1.7)
	*Lactobacillus*	2 (1.7)
	*Candida* spp and diphtheroid	1 (0.8)
Urine	No growth or non-pathogen	107 (87.7)
	*E. coli*	11 (9.0)
	*Klebsiella*	3 (2.5)
	Diphtheroid	1 (0.8)

### Between-group comparisons

Women with FGM type III had a significantly higher prevalence of positive urine culture growth compared with those with types I–II (20.4% vs 6.2%, p=0.024). A history of UTI and prior antibiotic use for UTI were also markedly more common in the type III group (95.9% vs 67.2% and 93.9% vs 68.8%, respectively; both p<0.001). In addition, abdominal pain was reported significantly more frequently among women with FGM type III (91.8% vs 60.9%, p<0.001). No statistically significant differences were observed between groups with respect to smoking status, daily coffee consumption, itching, vomiting, cloudy or offensive urine, haematuria, involuntary micturition or vaginal discharge (all p>0.05; table 3).

**Table 3:** Urinary tract infection and symptoms according to FGM type

Variable	Types I and II FGM (n=64,%)	Type III FGM (n=49,%)	P value
Urine culture growth			**0.024**
No growth	60 (93.8)	39 (79.6)	
Growth	4 (6.2)	10 (20.4)	
History of UTI			**<0.001**
No	21 (32.8)	2 (4.1)	
Yes	43 (67.2)	47 (95.9)	
History of antibiotic use for UTI			**0.001**
No	20 (31.3)	3 (6.1)	
Yes	44 (68.8)	46 (93.9)	
Smoking			0.785
No	62 (96.9)	47 (95.9)	
Yes	2 (3.1)	2 (4.1)	
Daily coffee consumption			0.372
1–2 cups	7 (10.9)	3 (6.1)	
Do not drink	57 (89.1)	46 (93.9)	
Itching			0.973
Absent	9 (14.1)	7 (14.3)	
Present	55 (85.9)	42 (85.7)	
Vomiting			0.848
Absent	63 (98.4)	48 (98.0)	
Present	1 (1.6)	1 (2.0)	
Abdominal pain			<0.001
Absent	25 (39.1)	4 (8.2)	
Present	39 (60.9)	45 (91.8)	
Cloudy urine			0.930
Absent	41 (64.1)	31 (63.3)	
Present	23 (35.9)	18 (36.7)	
Offensive urine			0.326
Absent	36 (56.3)	23 (46.9)	
Present	28 (43.8)	26 (53.1)	
Involuntary micturition			0.414
Absent	50 (78.1)	35 (71.4)	
Present	14 (21.9)	14 (28.6)	
Vaginal discharge			0.454
No	4 (6.3)	1 (2.0)	
White discharge	39 (60.9)	34 (69.4)	
Yellow, bad smelling	21 (32.8)	14 (28.6)	

FGM, female genital mutilation; UTI, urinary tract infection.

Women with FGM types III had significantly higher median scores across all urinary symptom domains compared with those with types I–II. Storage, voiding, incontinence, bladder pain, post-micturition symptoms and impaired ability to voluntarily stop urine flow were all significantly more severe in the type III group (all p<0.001). Similarly, symptom-related bother scores for storage, voiding, incontinence, post-micturition symptoms and pain were significantly higher among women with FGM type III (all p<0.001; table 4).

**Table 4:** Urinary symptom and bothering scores by FGM type

Score type	FGM types I and II median (IQR)	FGM type III median (IQR)	P value
Urinary scores			
Storage	2.0 (1.5–2.5)	4.0 (3.1–4.5)	<0.001
Voiding	1.9 (1.1–2.4)	4.0 (3.3–4.7)	<0.001
Incontinence	2.1 (1.3–2.9)	4.1 (3.4–4.9)	<0.001
Bladder pain	2.0 (1.2–2.7)	3.9 (3.2–4.8)	<0.001
Post-micturition	1.8 (1.0–2.4)	3.8 (3.1–4.6)	<0.001
Ability to stop urine flow	2.0 (1.3–2.6)	4.0 (3.3–4.8)	<0.001
Bothering scores			
Storage bothering	2.2 (1.5–3.0)	4.2 (3.5–5.0)	<0.001
Voiding bothering	1.7 (1.0–2.5)	4.0 (3.3–4.7)	<0.001
Incontinence bothering	2.0 (1.2–2.8)	4.1 (3.5–4.8)	<0.001
Post-micturition bothering	1.9 (1.1–2.6)	4.0 (3.2–4.8)	<0.001
Pain bothering	2.1 (1.4–2.9)	4.2 (3.5–5.0)	<0.001

P values in bold font (P < 0.05).

FGM, female genital mutilation.

### Regression analysis

The results of the univariable logistic regression analysis of clinical and genitourinary characteristics associated with FGM type III are presented in table 5. Women with FGM type III demonstrated significantly higher odds of urine culture growth (OR: 3.90, 95% CI 1.13 to 13.13, p=0.024), previous UTI history (OR: 11.48, 95% CI 2.54 to 51.84, p=0.002) and abdominal pain (OR: 7.21, 95% CI 2.31 to 22.53, p<0.001). A history of antibiotic use for UTI was inversely associated with genitourinary tract symptoms (OR: 0.14, 95% CI 0.04 to 0.52, p=0.003). No significant associations were observed for smoking, daily coffee consumption, itching, vomiting, cloudy urine, offensive urine, involuntary micturition or abnormal vaginal discharge (all p>0.05).

**Table 5:** Univariable logistic regression analysis of clinical and genitourinary characteristics associated with FGM type III

Parameters	OR (95%CI)	P value
Urine culture growth	3.9 (1.13 to 13.13)	0.024
History of UTI	11.48 (2.54 to 51.84)	0.002
History of antibiotic use for UTI	0.14 (0.04 to 0.52)	0.003
Smoking	1.32 (0.18 to 9.71)	0.786
Daily coffee consumption	0.53 (0.13 to 2.17)	0.378
Itching	0.98 (0.34 to 2.85)	0.973
Vomiting	1.31 (0.08 to 21.52)	0.849
Abdominal pain	7.21 (2.31 to 22.53)	<0.001
Cloudy urine	1.04 (0.48 to 2.24)	0.930
Offensive urine	1.45 (0.69 to 3.07)	0.327
Involuntary micturition	1.43 (0.61 to 3.37)	0.415
Vaginal white discharge	3.49 (0.37 to 32.80)	0.274
Vaginal yellow, bad smelling discharge	2.67 (0.27 to 26.40)	0.402

Reference category for FGM status was FGM types I and II

FGM, female genital mutilation; UTI, urinary tract infection.

The results of the univariable and multivariable logistic regression analyses evaluating the association between FGM type III and LUTS outcomes are presented in table 6. FGM type III was significantly associated with increased odds of more severe LUTS across all ICIQ-FLUTS domains. The strongest associations were observed for pain bothering, voiding symptoms and storage bothering. All urinary symptom and bother domains were significantly associated with FGM type III in the univariable logistic regression analyses (all p<0.001). After adjustment for the selected covariates, the association between FGM type III and all ICIQ-FLUTS symptom and bother domains remained statistically significant (all p<0.001).

**Table 6:** Association between FGM type III and LUTS outcomes

Parameters	OR (95%CI)	P value	AOR (95%CI)	P value
Storage score	2.41 (1.88 to 3.10)	<0.001	2.12 (1.58 to 2.86)	<0.001
Voiding score	2.76 (2.05 to 3.71)	<0.001	2.43 (1.72 to 3.42)	<0.001
Incontinence score	2.32 (1.79 to 3.01)	<0.001	1.94 (1.41 to 2.66)	<0.001
Bladder pain score	2.48 (1.89 to 3.25)	<0.001	2.08 (1.50 to 2.88)	<0.001
Post-micturition score	2.29 (1.74 to 3.00)	<0.001	1.88 (1.35 to 2.61)	<0.001
Ability to stop urine flow	2.57 (1.95 to 3.38)	<0.001	2.21 (1.61 to 3.03)	<0.001
Storage bothering	2.71 (2.02 to 3.64)	<0.001	2.37 (1.68 to 3.33)	<0.001
Voiding bothering	2.63 (1.98 to 3.50)	<0.001	2.29 (1.65 to 3.17)	<0.001
Incontinence bothering	2.54 (1.91 to 3.37)	<0.001	2.16 (1.55 to 3.00)	<0.001
Post-micturition bothering	2.46 (1.85 to 3.28)	<0.001	2.04 (1.46 to 2.84)	<0.001
Pain bothering	2.88 (2.11 to 3.93)	<0.001	2.58 (1.80 to 3.69)	<0.001

Reference category: FGM types I and II

Adjusted ORs were obtained from separate multivariable logistic regression models constructed for each LUTS outcome; candidate covariates were selected according to the modelling strategy described in the Statistical analysis section and were included to obtain adjusted estimates; they were not interpreted as independent risk factors

AOR, adjusted OR; FGM, female genital mutilation; LUTS, lower urinary tract symptoms.

## Discussion

In this study, among symptomatic Somali women with FGM who sought medical care, those with FGM type III had higher rates of culture-confirmed UTI and more severe LUTS than women with types I–II.

These findings represent a clinically relevant difference that strongly support preventive healthcare strategies aimed at the elimination of FGM and reduction of its associated urological complications. Our findings align with and extend previous research demonstrating increased urologic morbidity following FGM. Amin *et al* reported similar patterns of elevated LUTS prevalence in women with FGM, noting that symptom severity correlated with the extent of genital cutting.^15^ Similar to prior studies reporting increased urinary hesitancy, weak stream, nocturia and urinary incontinence in women with severe FGM.^16 17^ These findings are also consistent with a recently published study examining the association between emotional distress and LUTS among Somali migrant women with a history of FCM.^18^

The predominance of *E. coli* as the primary uropathogen in our study is consistent with global patterns of UTI aetiology.^19^ The increased UTI rates in women with extensive FGM likely reflect multiple interconnected mechanisms. FGM type III involves extensive tissue removal and creation of a small opening for urine and menstrual flow, leading to several pathophysiologic alterations that promote bacterial infection.^20^ First, the narrowed urethral opening can cause incomplete bladder emptying, creating post-void residual urine that serves as a bacterial growth medium.^21^ Second, the altered anatomy may cause turbulent urine flow patterns that facilitate bacterial ascension into the urinary tract.^22^ Chronic inflammation and scarring from repeated infections can further compromise normal voiding mechanics, creating a self-perpetuating cycle of urologic dysfunction.^23^ The significantly elevated LUTS scores across all domains in type III group support this mechanistic framework, demonstrating functional impairment in storage, voiding and continence mechanisms.

These findings carry important implications for healthcare providers serving populations affected by FGM. The near-universal history of UTIs in women with FGM type III (95.9%) suggests that routine urologic screening may be considered in this population. Early identification of asymptomatic bacteriuria may prevent progression to symptomatic infection and reduce the risk of complications such as pyelonephritis.

Healthcare systems in high-prevalence regions may consider implementing specialised FGM care protocols that include systematic UTI screening during routine gynaecologic visits, patient education regarding proper perineal hygiene and voiding habits, prompt evaluation of LUTS with validated assessment tools and multidisciplinary care teams, including urologists familiar with FGM-related complications.^24^

The substantial symptom burden documented in our study indicated that women with extensive FGM would benefit from comprehensive urologic evaluation rather than episodic UTI treatment alone. Conservative management strategies, including pelvic floor physiotherapy, bladder training and behavioural modifications, may provide symptomatic relief while avoiding potential surgical complications.^25^

From a public health perspective, these findings reinforce the urgent need for FGM prevention efforts while simultaneously addressing the healthcare needs of the estimated 200 million women and girls already affected by these practices.^1^ The concentration of severe complications in FGM type III highlights the particular vulnerability of women in regions where infibulation is culturally practised.

Healthcare system strengthening in affected regions should prioritise training for healthcare providers in FGM-related complications, development of culturally sensitive care protocols and integration of FGM care into existing reproductive health services. Community education programmes addressing UTI prevention and early recognition of symptoms may empower women to seek timely medical care.^26^

The findings of this study should be interpreted within the context of the study population. Our participants consisted of symptomatic women with FGM who sought care at a tertiary referral hospital and met predefined eligibility criteria. Therefore, these findings may not be generalisable to all women with FGM, particularly asymptomatic women or those with common medical comorbidities who were excluded from the study. Although these eligibility criteria improved the internal comparability of the study population by reducing potential confounding, they may have limited the external validity of the findings. Future population-based studies that include both symptomatic and asymptomatic women are warranted to better characterise the full spectrum of LUTS associated with FGM.

## Limitations

The limitations of this study should be considered when interpreting the findings. First, the cross-sectional design precludes establishing causal relationships between FGM type and LUTS severity. Second, the hospital-based recruitment strategy may have introduced selection bias, as women presenting to tertiary care facilities may experience more severe complications than the general population of women with FGM. Third, cultural and linguistic factors may have influenced symptom reporting and healthcare-seeking behaviour, potentially affecting the accuracy of LUTS assessment. Fourth, the absence of a non-FGM control group should be acknowledged as a limitation, as it restricts the ability to fully quantify the absolute risk associated with FGM exposure. In addition, UTIs were defined using a ≥10⁵ CFU/mL threshold; therefore, cases with lower colony counts (eg, ≥10³ CFU/mL) could not be assessed, potentially leading to an underestimation of UTI frequency.

Because the study population consisted of symptomatic, care-seeking women with FGM and excluded several common comorbidities, the findings should not be generalised to all women with FGM.

Although FGM typology was classified according to clinical examination findings, respondent bias associated with self-reported symptoms and questionnaire-based assessments cannot be excluded. The use of Likert-scale instruments may also introduce limitations, including subjective interpretation of response options, forced-choice constraints that may not fully reflect the true perceptions of participants and response biases such as social desirability and central tendency effects. Finally, the study focused primarily on clinically presenting cases, which may have underestimated asymptomatic infections. In addition, the outcome-specific, partly data-driven selection of candidate covariates may have resulted in residual confounding and model-dependent estimates. Future qualitative and longitudinal studies may help better characterise FGM-related urologic symptoms and barriers to healthcare access.

## Conclusion

This study found that, among symptomatic care-seeking women with FGM, type III was associated with greater LUTS burden, higher reported history of UTI and greater symptom-related distress compared with types I and II. These findings highlight the need for comprehensive clinical assessment and appropriate management of urinary symptoms in this patient population.

### Ethics approval and consent to participate

The study was approved by the Ethics and Research Committee of Mogadishu Somali Turkey Training and Research Hospital (permission number and date: 833/MSTH/15314, 7 September 2023). The study was performed in accordance with the principles and guidelines of the Declaration of Helsinki. Written informed consent to participate in the study was obtained from all participants prior to enrolment. As a considerable proportion of the participants were illiterate, informed consent was obtained from their legal representatives. Analysis and reporting of the results are in compliance with the Strengthening the Reporting of Observational Studies in Epidemiology (STROBE) checklist.

## Data Availability

Data are available upon reasonable request.
