# Supplementary material for: Association between female genital mutilation type and lower urinary tract symptoms among symptomatic Somali women: a cross-sectional study
Source: BMJ Open. 2026 Sep 2;16(9):e118031. doi: 10.1136/bmjopen-2026-118031 (PMC13548279; doi:10.1136/bmjopen-2026-118031)
Supplement: Supplementary file 1 [file bmjopen-2026-118031.reviewer_comments.pdf]

PEER REVIEW HISTORY

BMJ Open publishes all reviews undertaken for accepted manuscripts. Reviewers are asked to complete a checklist review form and are provided with free text boxes to elaborate on their assessment. These free text comments are reproduced below.

ARTICLE DETAILS

Title (Provisional)

Association Between Female Genital Mutilation Type and Lower Urinary Tract Symptoms Among Symptomatic Somali Women: A Cross-Sectional Study

Authors

Erkok, Umut; keleş, esra; Barut, Adil; Saglik, Bilgehan; Aksoy, Levent; Hirsi, Mohamed Omar; Mohamud, Said Mohamed; Omer, Zeynep; Kul, Gizem; Ceylan, Meryem

VERSION 1 - REVIEW

**Reviewer**	**1**
**Name**	**Ahmed, Wisal**
**Affiliation**	**University of Washington, Global Health Department**
**Date**	**13-Mar-2026**
**COI**	**None**

I read this study with interest.

Major comments:

The authors would need to justify/provide better rationale on w this knowledge gap matters given existent literature/systematic reviews and 2025 WHO recommendations on having all forms of FGM be screened for LUTS. What added knowledge will be generated on what is known now in course of clinical management? or from a programmatic point - will this change the interventions.

Statistical decision on grouping Type III and IV does not make sense as FGM typology is NOT graded by severity. It is descriptive only and severity depends on degree of injury. Sample size only is not sufficient and suggest authors to either compare type III which is distinct to all other forms instead given the sample size.

Discussion should include how the tool used compares with many other tools used before.

Discussion should consider latest literature to compare study

Limitations need to address quality assurance for typology verification, respondent bias.

Minor

Sample size estimate needs to be explained better

Ethical standards in clinical exam and sample handling need to be addressed

Consistency of terminology use - FGM vs FGM/C vs Circumcision (not recommended)

Some references need to be updated

Limitations on causality need to be revised.

Use of Mann Whitney test revised it is for ordinal values not continuous

Use of Likert scale limitations need to be addressed such as being subjective interpretation of response options, forced choices that may not reflect true opinions, and response biases such as social desirability or central tendency bias

**** The reviewer provided a marked copy with additional comments. Please contact the publisher for full details. ****

Reviewer 2

Name Llor, Carl

Affiliation University of Southern Denmark, Department of Public Health, General Practice.

Date 08-Apr-2026

COI None

This is a good observational study addressing an unexplored problem in some countries. I have some comments:

MAJOR COMMENTS:

Why did you consider UTIs only when the number of colony-forming units was greater than 10^5, rather than using the more current threshold of 10^3? This choice likely led to an underestimation of the number of positive UTIs. If these data are available, it would be helpful to revise the analysis accordingly.

Additionally, the manuscript does not report how outcomes varied depending on whether procedures were performed by trained midwives and nurses versus other professionals. Were there differences in the types of mutilations, rates of UTIs, or other outcomes depending on the provider? This information would be valuable.

Was this study approved by a Ethics Committee? I haven't read this.

MINOR COMMENTS:

Two sentences are repeated in the Introduction and should be corrected.

The sample size calculation is incomplete, and the relatively small sample size should be explained in more depth.

The subheading 'Participant Characteristics' could be shortened, as this information is already presented in Table 1.

VERSION 1 - AUTHOR RESPONSE

Reviewer 1

We sincerely thank Reviewer 1 for the valuable and detailed comments, which have significantly improved the quality of our manuscript.

Major comments

1. Rationale and clinical/programmatic relevance of the study
We appreciate this important comment. While current literature and the 2025 WHO recommendations support universal screening of all FGM types for LUTS, limited comparative evidence exists regarding symptom severity and infection risk across FGM types. Our study contributes by providing quantitative comparisons of LUTS severity and patient-reported bother across FGM types. Our findings demonstrate that more severe FGM types are associated with higher symptom burden and increased history of urinary tract infections. These results do not challenge existing screening recommendations but rather support risk-informed clinical prioritization. The manuscript has been revised accordingly.

2. FGM type grouping (Type III and IV)
We thank the reviewer for this important methodological concern. We acknowledge that FGM classification is descriptive rather than severity-based and that Type IV is heterogeneous. In the revised analysis, we have separated Type III from Type IV to ensure methodological consistency and avoid potential misclassification bias. Accordingly, comparisons are now presented as FGM Types I–II versus Type III, while Type IV is reported descriptively due to its limited sample size. The manuscript has been updated.

3. Comparison of tools and updated literature
We thank the reviewer for this suggestion. The Discussion section has been revised to include a more comprehensive comparison of the ICIQ-FLUTS-LF questionnaire with previously used instruments in the literature. Additionally, recent studies on LUTS in women with FGM have been incorporated to better contextualize our findings.

4. Limitations (typology verification and respondent bias)
We thank the reviewer for this important comment. The Limitations section has been revised to include clarification that FGM typology was determined through clinical examination, while acknowledging potential respondent bias associated with self-reported questionnaire data.

Minor comments

1. Sample size estimation
We thank the reviewer for this comment. A post-hoc power analysis was performed using G*Power (v3.1.9.7). The analysis suggested adequate statistical power (≥80%) to detect differences in urine culture positivity between groups. These details have been added to the Statistical Analysis section.

2. Ethical standards
We thank the reviewer for this comment. The manuscript has been revised to clearly state that ethical approval was obtained from the relevant Ethics Committee, and that all participants provided informed consent. All clinical and laboratory procedures were performed in accordance with the Declaration of Helsinki and institutional guidelines, ensuring patient confidentiality, privacy, and standardized sample handling.

3. Terminology consistency
We thank the reviewer for this comment. The manuscript has been revised to ensure full consistency by using the term "FGM" throughout, in accordance with WHO terminology guidelines. All other variations have been removed.

4. References updated
We thank the reviewer for this comment. The reference list has been updated with recent relevant literature.

5. Causality limitation
We thank the reviewer. The Limitations section has been revised to clearly state that the cross-sectional design does not allow causal inference between FGM type and LUTS.

6. Mann–Whitney U test clarification
We thank the reviewer for this important methodological comment. The Methods section has been revised to clarify that the Mann–Whitney U test was used for non-normally distributed continuous variables and ordinal outcomes, as appropriate.

7. Likert scale limitations
We thank the reviewer for this comment. The Limitations section has been expanded to include potential biases related to Likert-scale instruments, including subjective interpretation, forced-choice limitations, social desirability bias, and central tendency bias.

Reviewer 2

We sincerely thank Reviewer 2 for the positive evaluation and constructive suggestions.

Major comments

1. UTI definition (≥10^5 CFU/mL threshold)
We thank the reviewer for this important comment. In this study, UTI was defined using the ≥10^5 CFU/mL threshold, consistent with laboratory reporting standards at the time of data collection. We acknowledge that current guidelines increasingly support lower thresholds (≥10^3 CFU/mL), particularly in symptomatic patients. However, raw data below this

threshold were not available for re-analysis; therefore, sensitivity analysis using lower cut-offs could not be performed. This limitation has been added to the Discussion section.

2. Operator type and procedures
We thank the reviewer for this comment. All clinical procedures were performed by two specialist physicians under standardized conditions. There was no variability in provider allocation; therefore, no comparative analysis by operator type was applicable.

3. Differences by provider type
As all procedures were conducted by the same trained physicians and outcome data were primarily patient-reported (ICIQ-FLUTS-LF), stratified analysis by provider was not applicable.

4. Ethical approval
We thank the reviewer for this comment. Ethical approval, informed consent procedures, and compliance with the Declaration of Helsinki were obtained and are clearly stated in the revised manuscript.

Minor comments

1. Repeated sentences in Introduction
We thank the reviewer. The Introduction has been revised to remove duplication and improve clarity.

2. Sample size explanation
We thank the reviewer for this comment. The sample size estimation has been clarified using G*Power, and additional justification for subgroup sizes has been added to the Methods and Limitations sections.

3. Subheading simplification
We thank the reviewer. The section has been revised and streamlined as suggested.

Competing Interests

We thank both reviewers for this clarification. The statement indicating potential competing interests was included in error. The authors declare that there are no competing interests.

We once again thank the Editor and both reviewers for their valuable time and constructive feedback, which have significantly strengthened our manuscript.

VERSION 2 - REVIEW

Reviewer 2

Name Llor, Carl

Affiliation University of Southern Denmark, Department of Public Health, General Practice.

Date **29-May-2026**

COI

None.

Reviewer **3**

Name **Kurth, Tobias**

Affiliation **Charité Universitätsmedizin Berlin, Institute of Public Health**

Date **22-Jul-2026**

COI **TK reports having received research grants from the German Federal Joint Committee (G-BA) and personal compensation from the North-East German Society for Gynecological Oncology (NOGGO), AbbVie, the BMJ Group, and Frontiers Media S.A.**

In this cross-sectional study, Erkok and colleagues aimed to investigate lower urinary tract symptoms, urinary tract infection history, and symptom-related quality-of-life impact among Somali women with female genital mutilation/cutting (FGM) who presented with lower urinary tract symptoms, and to compare findings between women with FGM Types I–II and Type III. The topic is clinically and public-health relevant, and the use of a standardized lower urinary tract symptom instrument is a strength. However, several methodological and reporting issues limit the interpretability of the findings. I understand that this is a revised version and that I was not part of the first review. The authors may wish to consider the following comments.

1. The aim of the study should be clarified. It is not fully clear whether the authors intend the study to be descriptive, predictive, or causal. This distinction matters because each aim requires a different analytic framework and interpretation. The lack of clarity is also reflected in the multivariable model building. The authors should define the main aim more clearly and align the statistical analysis and interpretation accordingly.

2. The variable-selection strategy for the multivariable models should be reconsidered. Selecting variables based on statistical significance in univariable analyses is not recommended for confounding control or for building prediction models; see 10.1016/0895-4356(96)00025-X. Covariates should instead be selected based on clinical and causal reasoning, and the authors should clearly state which variables were included in each adjusted model and why. Given the modest sample size and small number of events for some outcomes, overfitting should also be considered.

3. I understand that sample size adequacy was raised in the previous review. However, a post-hoc power analysis is not very helpful for interpreting the findings and may be

misleading. The authors should instead focus on effect estimates and confidence intervals, and discuss imprecision where relevant (10.3899/jrheum.211115).

4. The study population should be described and interpreted more cautiously. The inclusion criteria were women aged ≥18 years presenting with lower urinary tract symptoms, with several exclusions, including pregnancy, diabetes, hypertension, coronary artery disease, renal or liver failure, and medications affecting lower urinary tract function. The findings therefore apply to a selected group of symptomatic, care-seeking women with FGM, not to women with FGM more broadly. This should be reflected consistently in the title, abstract, conclusions, and discussion. The exclusion of common comorbidities may improve internal comparability, but limits clinical generalizability.

5. I am concerned that the manuscript may invite a Table 2 fallacy. If the multivariable model is intended to estimate the association between FGM type and lower urinary tract outcomes, the adjusted estimates for covariates should not be interpreted as separate “independent risk factors” or causal effects. The role of each covariate in the model should be made clear. If the goal is adjustment, the interpretation should focus on the association of FGM type with the outcome after adjustment for selected covariates, not on the individual coefficients for all variables in the model.

6. The conclusions should be adjusted to the analytic framework. At present, the manuscript does not provide a causal framework to support causal inference about the effect of FGM Type III on lower urinary tract symptoms. Therefore, the conclusion should avoid causal wording such as “demonstrates” and “impact.” A more appropriate conclusion would state that, among symptomatic care-seeking women with FGM, Type III was associated with greater lower urinary tract symptom burden, higher reported history of urinary tract infection, and greater symptom-related distress.

VERSION 2 - AUTHOR RESPONSE

Response to Reviewers

Comment 1-.The aim of the study should be clarified. It is not fully clear whether the authors intend the study to be descriptive, predictive, or causal. This

distinction matters because each aim requires a different analytic framework and interpretation. The lack of clarity is also reflected in the multivariable

model building. The authors should define the main aim more clearly and align the statistical analysis and interpretation accordingly.

Response: We thank the reviewer for this important comment. We agree that the primary aim of the study should be stated more explicitly. The objective of this cross-sectional study was not to develop a prediction model or to make causal inferences, but rather to examine the association between FGM type and lower urinary tract symptoms among symptomatic

care-seeking women with FGM. Accordingly, we have revised the Introduction to clearly state this objective and have aligned the statistical analysis and interpretation with this analytic framework. Throughout the manuscript, we have avoided causal language and clarified that the multivariable logistic regression models were used to estimate the association between FGM type and LUTS outcomes after adjustment for potential confounding variables.

Comment 2.The variable-selection strategy for the multivariable models should be reconsidered. Selecting variables based on statistical significance in univariable analyses is not recommended for confounding control or for building prediction models; see 10.1016/0895-4356(96)00025-X. Covariates should instead be selected based on clinical and causal reasoning, and the authors should clearly state which variables were included in each adjusted model and why. Given the modest sample size and small number of events for some outcomes, overfitting should also be considered.

Response: We thank the reviewer for this important methodological comment. We acknowledge that selecting covariates solely on the basis of univariable statistical significance is not the preferred approach for confounder adjustment. In our study, however, the $p < 0.20$ threshold was used only to identify candidate variables for each outcome-specific model and was not the sole criterion for variable inclusion. Clinically relevant variables were also considered in model construction. Separate multivariable logistic regression models were developed for each LUTS domain, and we have revised the Methods section to clearly describe this modelling strategy. We now explicitly report the covariates included in each adjusted model and clarify that they were selected to control for potential confounding. Given the modest sample size and the limited number of events for some outcomes, we used parsimonious models and restricted the number of adjustment variables to minimise the risk of overfitting. No automated stepwise variable-selection procedure was used.

Comment 3. I understand that sample size adequacy was raised in the previous review. However, a post-hoc power analysis is not very helpful for interpreting

the findings and may be misleading. The authors should instead focus on effect estimates and confidence intervals, and discuss imprecision where

relevant (10.3899/jrheum.211115).

Response: The post-hoc power analysis was originally included in response to a previous reviewer's request regarding sample size adequacy. Following the current reviewer's recommendation, we have removed the post-hoc power analysis from the manuscript and

now rely on the reported effect estimates and 95% confidence intervals to reflect the magnitude and precision of the observed associations.

Comment 4. The study population should be described and interpreted more cautiously. The inclusion criteria were women aged ≥18 years presenting with lower urinary tract symptoms, with several exclusions, including pregnancy, diabetes, hypertension, coronary artery disease, renal or liver failure, and medications affecting lower urinary tract function. The findings therefore apply to a selected group of symptomatic, care-seeking women with FGM, not to women with FGM more broadly. This should be reflected consistently in the title, abstract, conclusions, and discussion. The exclusion of common comorbidities may improve internal comparability, but limits clinical generalizability.

Response: We thank the reviewer for this important comment. We agree that our study population represents a selected group of symptomatic, care-seeking women with FGM rather than all women with FGM. Accordingly, we have clarified this in the Methods section and revised the title, abstract, discussion, and conclusions to ensure that the findings are interpreted within the appropriate clinical context. We have also expanded the limitations section to acknowledge that the exclusion of common comorbidities improves internal comparability but may limit the generalisability of the findings.

Comment 5. I am concerned that the manuscript may invite a Table 2 fallacy. If the multivariable model is intended to estimate the association between FGM

type and lower urinary tract outcomes, the adjusted estimates for covariates should not be interpreted as separate “independent risk factors” or causal

effects. The role of each covariate in the model should be made clear. If the goal is adjustment, the interpretation should focus on the association of

FGM type with the outcome after adjustment for selected covariates, not on the individual coefficients for all variables in the model.

Response: We thank the reviewer for this important observation. We agree that the purpose of the multivariable models was to estimate the association between FGM type and LUTS outcomes after adjustment for potential confounding variables, rather than to identify independent risk factors or infer causal effects for the adjustment variables. Accordingly, we have clarified the purpose of the multivariable models in the Methods section, revised the Results to focus on the adjusted association between FGM type and LUTS outcomes, and added a footnote to Table 6 stating that the covariates were included solely for confounder adjustment and should not be interpreted as independent risk factors.

Comment 6. The conclusions should be adjusted to the analytic framework. At present, the manuscript does not provide a causal framework to support causal

inference about the effect of FGM Type III on lower urinary tract symptoms. Therefore, the conclusion should avoid causal wording such as

“demonstrates” and “impact.” A more appropriate conclusion would state that, among symptomatic care-seeking women with FGM, Type III was

associated with greater lower urinary tract symptom burden, higher reported history of urinary tract infection, and greater symptom-related distress.

Response: We thank the reviewer for this valuable comment. We agree that our cross-sectional study does not permit causal inference. Accordingly, we have revised the Conclusion to avoid causal language such as "demonstrates" and "impact". The revised conclusion now focuses on the observed associations and explicitly limits the interpretation to symptomatic care-seeking women with FGM, consistent with the study design and analytic framework.

VERSION 3 - REVIEW

**Reviewer**	**3**
**Name**	**Kurth, Tobias**
**Affiliation**	**Charité Universitätsmedizin Berlin, Institute of Public Health**
**Date**	**06-Aug-2026**
**COI**	

I thank the authors for their careful responses and revisions. My only remaining suggestion concerns the variable-selection strategy. Confounding is a causal rather than a statistical concept, and adjustment variables should, therefore, be selected based on substantive knowledge of the presumed causal structure rather than their univariable association with the outcome. Restricting the number of covariates may reduce model instability, but it does not ensure adequate confounding control. The authors should either reconsider the adjustment strategy or acknowledge that the outcome-specific, partly data-driven selection of covariates may have resulted in residual confounding and model-dependent estimates.

VERSION 3 - AUTHOR RESPONSE

Reviewer 3
Comment:
I thank the authors for their careful responses and revisions. My only remaining suggestion concerns the variable-selection strategy. Confounding is a causal rather than a statistical concept, and adjustment variables should, therefore, be selected based on substantive knowledge of the presumed causal structure rather than their univariable association with the outcome. Restricting the number of covariates may reduce model instability, but it does not ensure adequate confounding control. The authors should either reconsider the adjustment strategy or acknowledge that the outcome-specific, partly data-driven selection of covariates may have resulted in residual confounding and model-dependent estimates.
Response:
We sincerely thank the reviewer for this insightful methodological comment. We agree that confounding is fundamentally a causal concept and that statistical variable selection alone cannot ensure complete control of confounding.
In the present study, a $p<0.20$ threshold was used as a pragmatic screening criterion to identify candidate covariates for the multivariable logistic regression models. This approach was adopted to construct parsimonious models while reducing the risk of model instability given the available sample size. To improve transparency, we have revised the Statistical Analysis section to explicitly clarify the rationale for this modelling strategy.
Furthermore, in accordance with the reviewer's suggestion, we have expanded the Limitations section to acknowledge that the outcome-specific, partly data-driven selection of candidate covariates may have resulted in residual confounding and model-dependent estimates.
We believe these revisions improve the transparency of our analytical approach while preserving the original analyses, results, and overall interpretation of the study findings.
